# Millipede Dermatitis Mimicking Pediatric Snake Envenomation: A Case Report

**DOI:** 10.7759/cureus.110733

**Published:** 2026-06-12

**Authors:** Bradley M Golden, Nicole Fuller, Bryan Turner, Gary Prusky Grinberg

**Affiliations:** 1 Emergency Medicine, Augusta University Medical College of Georgia, Augusta, USA; 2 Pediatric Emergency Medicine, Augusta University Health, Augusta, USA; 3 Emergency Medicine, Augusta University Health, Augusta, USA

**Keywords:** benzoquinone pigmentation, cutaneous toxicology, emergency medicine, millipede dermatitis, pediatric dermatology, snakebite mimic

## Abstract

Millipede dermatitis is an uncommon clinical presentation caused by exposure to defensive secretions released by millipedes, often resulting in striking cutaneous discoloration that may mimic more serious conditions such as envenomation, infection, vasculitis, or tissue necrosis. We present the case of a previously healthy four-year-old male who developed acute black discoloration and mild pain involving the plantar surfaces of the right toes after playing barefoot outdoors. Physical examination demonstrated sharply demarcated black discoloration with surrounding erythema and mild edema while preserving neurovascular function and sparing the interdigital spaces. Initial concern for possible snake envenomation prompted empiric administration of CroFab and clindamycin prior to transfer. Laboratory evaluation, including complete blood count, coagulation studies, fibrinogen, creatine kinase, and metabolic testing, was unremarkable. Further history later revealed exposure to a black-and-yellow millipede, and the discoloration partially improved with alcohol swabbing, confirming exogenous pigmentation rather than tissue necrosis. The patient was managed conservatively with supportive care and experienced complete resolution without complications. This case highlights the importance of recognizing millipede dermatitis as a benign mimic of more serious toxicologic and infectious conditions in pediatric patients. Increased awareness may help avoid unnecessary antivenom administration, invasive interventions, hospital transfers, and healthcare costs.

## Introduction

Millipede dermatitis is an uncommon dermatologic condition resulting from contact with defensive secretions released by millipedes (class Diplopoda) [1]. Although millipedes are nonvenomous arthropods, many species secrete irritant compounds including benzoquinones, phenols, benzaldehyde, and hydrogen cyanide derivatives when threatened or crushed against the skin [2]. These chemicals may produce localized erythema, pain, blistering, chemical burn-like injury, and prolonged hyperpigmentation [3].

The striking appearance of these lesions may mimic more serious pathology, including cellulitis, vasculitis, necrotic arachnidism, thermal injury, and snake envenomation [4]. Pediatric cases may be particularly challenging due to limited exposure history and difficulty obtaining reliable symptom characterization. Several reports have described dramatic discoloration and plaque formation following millipede exposure, occasionally resulting in extensive diagnostic evaluation and unnecessary treatment [5,6].

We report a case of pediatric millipede dermatitis initially concerning for snake envenomation due to acute black discoloration of the toes and associated pain, ultimately diagnosed following subspecialty evaluation.

## Case presentation

**Figure 1:**
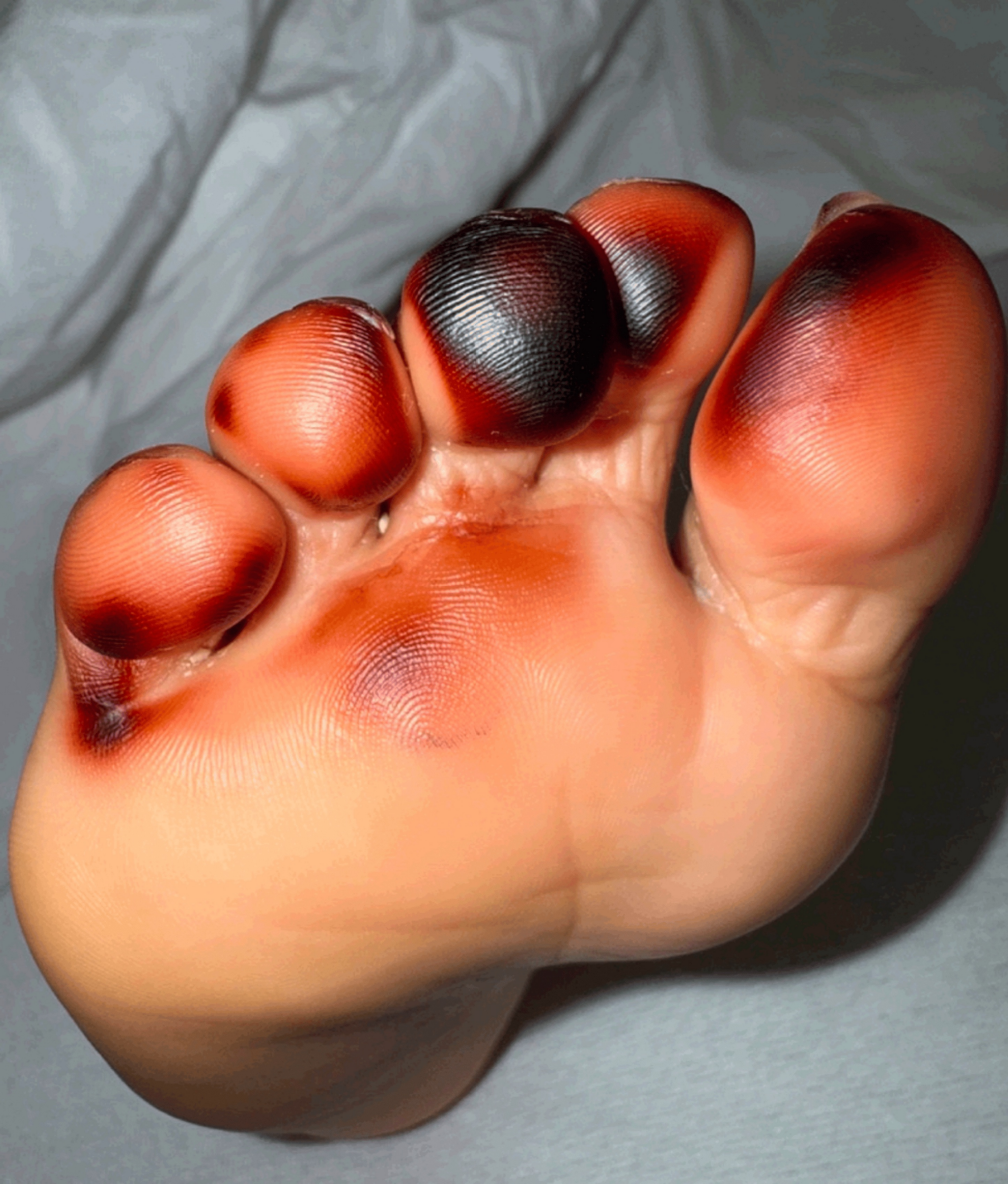
Plantar discoloration of the right toes secondary to millipede dermatitis Plantar view of the right foot demonstrating sharply demarcated black discoloration involving multiple toes and the distal plantar forefoot following suspected millipede exposure. The interdigital spaces are relatively spared, with no blistering, ulceration, or tissue necrosis identified. The striking pigmentation pattern initially raised concern for envenomation or ischemic injury prior to recognition of millipede-associated chemical dermatitis.

A previously healthy four-year-old male presented to the emergency department with acute discoloration and mild pain involving the toes of the right foot after playing barefoot outdoors the previous evening. The family initially suspected an insect bite; however, no puncture wound, bleeding, or immediate symptoms were noted at the time of exposure. By the following day, the patient developed progressive black discoloration of the plantar surfaces of the toes, accompanied by mild discomfort, prompting an emergency evaluation (Figure 1).

On examination, the patient appeared well and was in no acute distress. Vital signs were within normal limits for age. Physical examination demonstrated sharply demarcated black discoloration involving the plantar surfaces of all right toes with surrounding mild erythema and edema. The interdigital spaces were notably spared. There was no warmth, blistering, ulceration, drainage, crepitus, or skin breakdown. Neurovascular examination remained intact with preserved capillary refill, palpable distal pulses, and normal motor function. No proximal streaking or lymphangitic changes were identified.

Given the dramatic discoloration and concern for possible envenomation or evolving tissue injury, the patient underwent laboratory evaluation and received empiric CroFab and clindamycin prior to definitive diagnosis. Laboratory testing demonstrated a normal white blood cell count (7.2 thous/mm³), normal inflammatory markers with a C-reactive protein <0.020 mg/dL, normal creatine kinase of 178 U/L, normal coagulation studies, including PT 12.6 seconds and INR 1.1, and a normal D-dimer of <150 ng/mL. Fibrinogen was mildly decreased at 163 mg/dL. Additional laboratory findings are summarized in Table 1.

**Table 1:** Laboratory findings on presentation at the emergency department Results demonstrated no significant leukocytosis, inflammatory response, coagulopathy, or evidence of systemic tissue injury. Mild thrombocytosis and decreased fibrinogen were noted, while creatine kinase, C-reactive protein, D-dimer, and coagulation studies remained within normal limits.

Laboratory Test	Patient Value	Reference Range
White Blood Cell Count (WBC)	7.2 thous/mm³	4.5–11.0 thous/mm³
Red Blood Cell Count (RBC)	4.36 million/mm³	4.00–5.30 million/mm³
Hemoglobin	11.9 g/dL	11.0–16.0 g/dL
Hematocrit	34.90%	32.0–45.0%
Mean Corpuscular Volume (MCV)	80.1 fL	80.0–98.0 fL
Mean Corpuscular Hemoglobin (MCH)	27.4 pg	27.0–33.0 pg
Mean Corpuscular Hemoglobin Concentration (MCHC)	34.2 g/dL	32.0–36.0 g/dL
Red Cell Distribution Width (RDW)	13.20%	11.5–18.0%
Platelet Count	529 thous/mm³	150–400 thous/mm³
Mean Platelet Volume (MPV)	6.4 fL	7.4–10.4 fL
Neutrophils	52.90%	20.0–55.0%
Lymphocytes	31.70%	30.0–65.0%
Monocytes	9.60%	2.0–14.0%
Eosinophils	1.00%	0.0–5.0%
Basophils	1.90%	0.0–1.0%
Absolute Neutrophil Count	3.9 thous/mm³	1.8–7.8 thous/mm³
Absolute Lymphocyte Count	2.4 thous/mm³	1.0–2.8 thous/mm³
Absolute Monocyte Count	0.7 thous/mm³	0.0–0.8 thous/mm³
Absolute Eosinophil Count	0.1 thous/mm³	0.0–0.5 thous/mm³
Creatine Kinase (CK)	178 U/L	32–294 U/L
Prothrombin Time (PT)	12.6 sec	10.3–13.3 sec
International Normalized Ratio (INR)	1.1	0.9–1.1
D-dimer	<150 ng/mL (D-DU)	0–230 ng/mL
Lipase	14 U/L	6–51 U/L
C-Reactive Protein (CRP)	<0.020 mg/dL	0.000–0.500 mg/dL
Fibrinogen	163 mg/dL	200–400 mg/dL

Further history obtained during evaluation revealed possible exposure to a millipede while outdoors. When shown pictures of various insects and animals, the patient inconsistently identified several organisms, including a snake; however, the morphology and sharply localized discoloration pattern were ultimately felt to be most consistent with millipede-associated chemical dermatitis. The patient was managed conservatively with supportive care and observation. The discoloration gradually improved without evidence of necrosis, secondary infection, or long-term complications.

## Discussion

Millipedes are nonvenomous arthropods that rely on chemical defense mechanisms for protection against predators [1]. Depending on the species involved, defensive secretions may contain benzoquinones, quinones, phenols, benzaldehyde, and hydrogen cyanide compounds capable of producing localized dermatologic injury [2]. Exposure typically occurs when the organism is crushed against exposed skin, allowing toxin deposition onto the epidermis [7].

Clinical manifestations vary depending on toxin concentration, duration of contact, and individual sensitivity. Reported findings include a burning sensation, erythema, blistering, vesiculation, brown-black pigmentation, and chemical burn-like lesions [5,7]. Hyperpigmentation may persist for days to weeks before spontaneous resolution [8]. Prior reports have described lesions mistaken for vascular compromise, necrosis, and infectious processes due to their dramatic appearance [5,6].

In this case, sharply demarcated black discoloration involving multiple toes raised concern for possible snake envenomation. Pediatric evaluation of suspected envenomation can be challenging when the exposure event is unwitnessed, or the history is unreliable. The absence of puncture wounds, progressive edema, coagulopathy, systemic toxicity, or tissue destruction ultimately made pit viper envenomation less likely. Nevertheless, concern for occult envenomation prompted empiric CroFab administration prior to transfer.

This case highlights several distinguishing features of millipede dermatitis that may assist emergency physicians in avoiding unnecessary intervention. Millipede-associated lesions are often sharply demarcated, nonprogressive, and confined to areas of direct contact [4]. Interdigital sparing may occur when secretions contact only exposed skin surfaces. Systemic toxicity is generally absent, and laboratory studies are typically normal [3]. Recognition of these features may reduce unnecessary antibiotic exposure, antivenom administration, interfacility transfer, and healthcare utilization.

Management of millipede dermatitis is primarily supportive and includes cleansing of the affected area, symptomatic pain control, and reassurance [9,10]. Prognosis is excellent, with most cases resolving spontaneously without permanent sequelae [6].

## Conclusions

Millipede dermatitis is an uncommon but important mimic of pediatric snake envenomation and other serious dermatologic conditions. Characteristic, sharply demarcated discoloration following outdoor exposure, particularly in the absence of systemic toxicity or progressive tissue injury, should prompt consideration of arthropod toxin exposure. Familiarity with this entity may help emergency physicians avoid unnecessary antivenom administration, antibiotic therapy, hospital transfer, and invasive evaluation while providing appropriate supportive management and reassurance.
